# Iron limitation of kelp growth may prevent ocean afforestation

**DOI:** 10.1038/s42003-023-04962-4

**Published:** 2023-06-06

**Authors:** Ellie R. Paine, Philip W. Boyd, Robert F. Strzepek, Michael Ellwood, Elizabeth A. Brewer, Guillermo Diaz-Pulido, Matthias Schmid, Catriona L. Hurd

**Affiliations:** 1 Institute for Marine and Antarctic Studies, University of Tasmania, Hobart, TAS 7001 Australia; 2 Australian Antarctic Program Partnership (AAPP), Institute for Marine and Antarctic Studies, University of Tasmania, Hobart, TAS Australia; 3 Research School of Earth Sciences, The Australian National University, Canberra, ACT 0200 Australia; 4 CSIRO Oceans and Atmosphere, Castray Esplanade, Hobart, TAS 7001 Australia; 5 School of Environment and Science, Coastal and Marine Research Centre, and Australian Rivers Institute—Coast and Estuaries, Nathan Campus, Griffith University, Brisbane, QLD 4111 Australia; 6 Trinity College Dublin, University of Dublin, Dublin, Ireland; 7 School of Biological and Chemical Sciences, University of Galway, Galway, Ireland

**Keywords:** Plant physiology, Carbon cycle

## Abstract

Carbon dioxide removal (CDR) and emissions reduction are essential to alleviate climate change. Ocean macroalgal afforestation (OMA) is a CDR method already undergoing field trials where nearshore kelps, on rafts, are purposefully grown offshore at scale. Dissolved iron (dFe) supply often limits oceanic phytoplankton growth, however this potentially rate-limiting factor is being overlooked in OMA discussions. Here, we determine the limiting dFe concentrations for growth and key physiological functions of a representative kelp species, *Macrocystis pyrifera*, considered as a promising candidate for OMA. dFe additions to oceanic seawater ranging 0.01-20.2 nM Fe′ ‒ Fe′ being the sum of dissolved inorganic Fe(III) species ‒ result in impaired physiological functions and kelp mortality. Kelp growth cannot be sustained at oceanic dFe concentrations, which are 1000-fold lower than required by *M. pyrifera*. OMA may require additional perturbation of offshore waters via dFe fertilisation.

## Abstract (web summary)

Laboratory experiments with the macroalga *Macrocystis pyrifera* suggest that insufficient iron availability in the open ocean presents a major challenge to growing kelp for the purpose of sequestering carbon dioxide through ocean afforestation.

## Introduction

Ocean macroalgal afforestation (OMA) is one of more than 30 marine techniques proposed as suitable candidates for carbon dioxide (CO_2_) removal to mitigate climate change caused by increasing atmospheric CO_2_ levels^1,2^. OMA is based on the purposeful introduction of kelps (marine macroalgae or seaweed, order Laminariales) into the open ocean, where they grow on stationary seaweed farms or transit offshore attached to raft-like structures^1,3^. Kelps form dense underwater beds (‘forests’) in temperate, coastal marine ecosystems, providing habitat and food for higher trophic levels, and essential nitrogen and carbon cycling services. They take up CO_2_ from seawater and ‘fix it’ into organic matter through photosynthesis, yet whether or not this process leads to carbon sequestration (i.e., secure storage > 100 years^2,4^) is difficult to quantify^4^. Regardless, proponents of OMA consider that increasing the biomass of nearshore seaweeds by open-ocean colonisation will increase carbon sequestration^1,5^. Previous OMA research has determined geographical regions with sufficient macronutrient concentrations to sustain seaweed growth^6^. However, essential trace metals for photosynthesis, such as dFe, limit phytoplankton primary production in much of the open ocean^7^ and have been a major omission in the OMA debate.

Iron is the most studied trace element in the ocean with a significant influence on the functioning of the biological carbon cycle due to its crucial role in setting the rates of enzymatic activity of algal photosynthesis and nitrogen uptake^8,9^. Concentrations of dFe used in this study are expressed as Fe′ which is the sum of dissolved inorganic Fe(III) species, a proxy that best mimics dFe bioavailability. Concentrations of dFe vary with locale and depth, and dFe availability limits primary productivity in one-third of the global ocean^10^. This scarcity results in areas termed high nutrient and low chlorophyll (HNLC) where macronutrient inventories such as for nitrate (NO_3_^−^) can only be partially utilised^7,11,12^. In the coastal ocean, however, rivers, atmospheric inputs and sediments are significant, and often ongoing, sources of dFe, with concentrations ranging from ~0.1 to ~500 nM depending on fluvial inputs^13,14^. Coastal waters (defined here as waters extending from the shoreline to the outer edge of the continental margin) also have a higher concentration of bioavailable dFe compared to the open ocean due to increased concentrations of dFe binding ligands, such as humic and fulvic acids from sediment margins and riverine sources^15,16^. Consequently, algae which live in this coastal region are typically dFe-replete^17,18^.

For seaweeds, tissue iron content and its relationship to electron transport and pigment content is well documented for many species^11,17,19–24^; however, the role of seawater dFe concentrations for key physiological functions of seaweed—growth, dissolved organic carbon (DOC) production, photosynthesis and nitrogen metabolism—has not previously been studied and is a knowledge gap in OMA discussions^8,11^. However, there are likely many parallels with the multifaceted role that iron plays in other photosynthetic organisms, including oceanic phytoplankton^18,25^. For example, up to half of the dFe in photoautotrophs is used for photosynthesis—primarily electron transport between photosystem II (PSII) and photosystem I (PSI)^9,26^. dFe is essential for the synthesis of algal pigments, respiration and nitrogen assimilation with both NO_3_^−^ and nitrite (NO_2_^−^) reductases containing iron-rich haem groups^9,17,26–28^. The cumulative effect of iron-mediated regulation of these physiological pathways ultimately controls phytoplankton growth. For oceanic phytoplankton, dFe concentration regulates the release of DOC, a major global carbon pool (660 Pg C per annum)^29^. Phytoplankton DOC production increases with dFe limitation as an energy dissipation mechanism linked to nutrient availability^30^. Clearly, dFe has the potential to exert major influences on multiple seaweed physiological pathways.

Here, we investigated the influence of a gradient of seven Fe′ concentrations, 0.01, 1.85, 4.67, 9.56, 20.2, 45.2 and 120 nM (equivalent to dFe additions of 0.01–40 μM), on the growth and physiology of *M. pyrifera*. The Fe′ gradient straddled environmentally relevant concentrations for coastal and open oceans along with higher concentrations needed to fully describe the physiological relationships between various metrics and Fe′, similar to experiments for phytoplankton^31,32^, in order to explore the Fe′ requirements of *M. pyrifera*.

Our study species was the giant kelp, *Macrocystis pyrifera* (phylum Ochrophyta, order Laminariales), as it has an extensive geographic range, a high growth rate (~ 2% increase per day of the foliar standing crop), a high capacity to take up nutrients from seawater and, because of these traits, is the species of choice for many planned OMA endeavours^6,33^. To determine the Fe′ requirements of *M. pyrifera*, we performed physiological assays to measure growth, DOC production, photosynthesis, respiration, chlorophyll pigment content, maximum photochemical efficiency of photosystem II (F_*v*_/F_*m*_), tissue carbon and nitrogen, C:N ratios and soluble tissue NO_3_^−^. Seawater for experimental treatments was collected at a site (~ 47°S, 141°E) in the Southern Ocean (Supplementary Fig. 1), where Fe′ typically ranges from 0.11 to 0.72 pM in the seasonal surface mixed layer^8^. All other essential macro- and micro-nutrients for *M. pyrifera* growth were replete using Aquil medium nutrient additions to natural ocean seawater (buffered with 100 μM ethylenediaminetetraacetic acid (EDTA)) and contained 0.01 nM background Fe′.

## Results

### Iron limitation of *M. pyrifera* growth in the open ocean

**Fig. 1:**
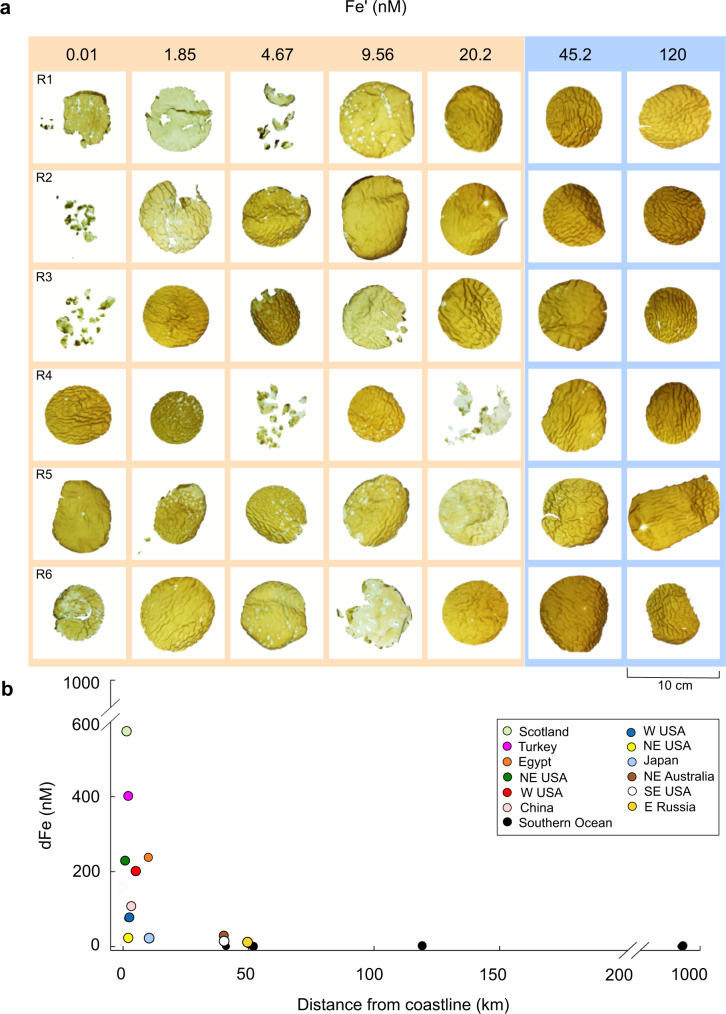
Condition of *M. pyrifera* replicates as a result of Fe′ concentrations and natural concentrations of dFe along a coastal to open-ocean gradient. **a** Photographs of individual discs of *M. pyrifera* replicates (*n* = 6, labelled R1-R6) at the end of a 14-day Fe′ experiment (0.01–120 nM). Coloured panels are used to represent the disparity between healthy replicates at 45.2–120 nM Fe′ (blue panel) and those displaying symptoms of physiological stress and mortality ≤ 20.2 nM Fe′ (orange panel). Inorganic Fe (Fe′) values in the upper panel correspond to total dFe concentrations of 0, 1, 2.5, 5, 10, 20 and 40 μM for each treatment ( + 7.25 nM background dFe in nutrient-spiked seawater). **b** Plot of dFe concentrations (nM) against distance offshore (km). Black circles denote dFe concentrations collected on GEOTRACES-SR3 Southern Ocean voyage^35^ as an example of the dFe concentrations observed uniformly in the open ocean (0.1–0.6 nM)^8,36^ and coloured circles depict dFe concentrations collated from a comprehensive literature search on coastal sites (see Supplementary Data 1). For comparison, the dFe concentration of 1000 nM equates to 1.85 nM Fe′.

At concentrations ≤20.2 nM Fe′, *M. pyrifera* displayed symptoms of multifaceted physiological stress and mortality, with visible tissue degradation and fragmentation of replicates in all treatments, whereas at 45.2 and 120 nM Fe′, all replicates were highly pigmented, intact and had surface corrugations typical of healthy blades^34^ (Fig. 1a).

To provide a wider environmental context for the findings from Fig. 1a, we plotted observed dFe concentrations (nM) in surface waters with distance (km) offshore (Fig. 1b). dFe from between 50 and 200 km offshore (black circles) was measured along GEOTRACES-SR3 transect from Tasmania to Antarctica^35^. Published dFe concentrations between 0-50 km from the coast are collated in Fig. 1b. It is not possible to report Fe′ for these studies as the additional data required for such calculations is not available (see Supplementary Fig. 2). Typically, a range of dFe of 0.1–0.6 nM would equate to an Fe′ of 0.11–1.49 pM (see Supplementary Equation). In our compilation, dFe concentrations decrease sharply offshore (Fig. 1b) ranging from 573 nM (Thurso Bay, Scotland, enriched by a riverine source^14^) to 0–0.327 nM (Southern Ocean). For comparison, the dFe concentration of 1000 nM equates to 1.85 nM Fe′. These low dFe concentrations in oceanic surface waters are observed uniformly across the globe (< 0.2 nM and average 0.07 nM)^36^. When compared with the Fe′ requirements for *M. pyrifera* (≥ 45.2 nM, Fig. 1a), the concentrations of Fe′ available offshore (0–1.49 pM^8,36^) are at least 1000-fold lower than required to sustain healthy growth (Fig. 1b).

### Relationship between dFe concentration, growth and DOC release

**Fig. 2:**
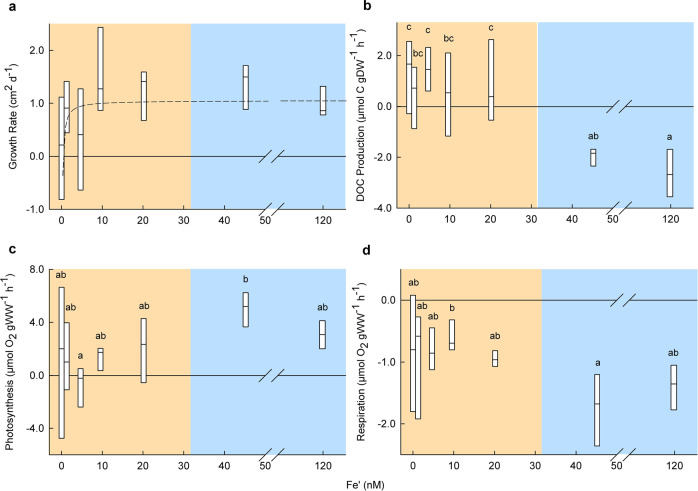
Fe′ concentrations drive rates of growth, photosynthesis, respiration and DOC production for *M. pyrifera*. **a**–**d** Influence of Fe′ concentration on *M. pyrifera* showing, **a** growth, with a modelled rectangular hyperbola (adjusted *R*^2^ = 0.122), **b** DOC production was observed at ≤ 20.2 nM Fe′ but not observed for 45.2–120 nM Fe′ (one-way ANOVA, *P* < 0.01, *F* value = 8.473, df = 6). The negative DOC flux at 45.2–120 nM Fe′ likely represents consumption by the seaweed biome^72^. **c** Increased net photosynthetic rates and **d** respiration rates at ≥ 45.2 nM compared to ≤ 9.56 nM Fe′ (one-way ANOVAs, photosynthesis *P* = 0.057, *F* value = 2.285, df = 6 and respiration *P* = 0.047, *F* value = 2.406, df = 6). Boxes show minimum, 1^st^ quartile, median, 3^rd^ quartile and maximum data values (*n* = 6). Note no box whiskers, as the lower quartile is equal to the minimum value, and the upper quartile is equal to the maximum value. For significant results, Fe′ concentrations displaying the same letter were not significantly different in post hoc tests. The disparity between healthy replicates observed in Fig. 1a (45.2–120 nM Fe′) and those with symptoms of physiological stress (≤ 20.2 nM Fe′) is indicated by blue and orange panels, respectively. For full statistical results and post hoc tests see Supplementary Data 2 and 3.

We used the findings from multiple physiological assays to understand the mechanisms that resulted in poor condition of *M. pyrifera* under concentrations of ≤ 20.2 nM Fe′ (Fig. 1a). Growth (cm^2^ d^−1^) of *M. pyrifera* increased with Fe′ concentration and although not statistically significant (*P* = 0.34), the relationship was best modelled using a rectangular hyperbola (Adjusted *R*^2^ = 0.122) which saturated at > 9.56 nM (Fig. 2a). At concentrations of ≤ 20.2 nM Fe′, there was a substantial increase in the amount of DOC produced (0.43–1.56 μmol C gDW^−1^ h^−1^), which was not evident ≥ 45.2 nM Fe′ (Fig. 2b). When DOC production is considered for individual kelp replicates (Fig. 1a, replicates labelled R1-R6), higher rates are observed for those with tissue disintegration, indicating that fragmentation of kelp blades enhanced DOC release (Supplementary Fig. 3)^37^. At ≥ 45.2 nM Fe′ any DOC released by *M. pyrifera* was rapidly metabolised by bacteria yielding negative DOC production rates in these incubations (Fig. 2b).

### Influence of dFe supply on key physiological functions

**Fig. 3:**
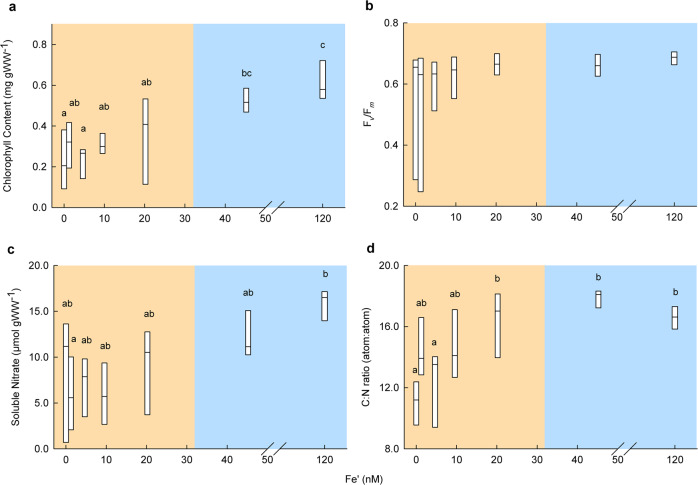
Fe′ concentrations drive photophysiology, nitrate storage and tissue carbon for *M. pyrifera*. **a**–**d** Influence of Fe′ concentration on *M. pyrifera* showing, **a** increased total chlorophyll content at 120 nM Fe′ compared to 0.01–1.85 nM Fe′ (one-way ANOVAs, chlorophyll *P* < 0.01, *F* value = 7.625, df = 6), **b** maximum photochemical efficiency of PSII (F_*v*_/F_*m*_) using PAM fluorometry, **c** increased soluble tissue content of nitrate (NO_3_^-^) at 120 nM compared to ≤ 9.56 nM Fe′ (one-way ANOVA, *P* = 0.02, *F* value = 2.911, df = 6) and **d** higher tissue carbon to nitrogen ratio at ≥ 45.2 nM compared to 0.01 nM Fe′ (one-way ANOVA, *P* < 0.01, *F* value = 9.232, df = 6). Boxes show minimum, 1^st^ quartile, median, 3^rd^ quartile and maximum data values (*n* = 6). Note no box whiskers, as the lower quartile is equal to the minimum value, and the upper quartile is equal to the maximum value. For significant results, Fe′ concentrations displaying the same letter were not significantly different in post hoc tests. Coloured panels are applied to represent the disparity between healthy replicates at 45.2–120 nM Fe′ (blue panel) and those ≤ 20.2 nM Fe′ (orange panel). For statistical results and post hoc tests see Supplementary Data 2 and 3.

Photosynthesis and respiration were both highly variable at concentrations ≤ 20.2 nM Fe′ yet were elevated above ≥ 45.2 nM Fe′ treatments (photosynthesis ~ 5700% greater at 45.2 than 4.67 nM Fe′ and respiration ~ 195% greater at 45.2 than 9.56 nM Fe′; Fig. 2c, d). Carbon to nitrogen ratios (C:N) of *M. pyrifera* were reduced at concentrations ≤ 20.2 nM Fe′, indicating carbon fixation and incorporation into kelp tissue was limited by Fe′ availability, resulting in the observed, substantial DOC release (Figs. 2b and 3a–d). Total chlorophyll pigments were higher in the 120 nM treatment compared to those at 0.01–4.67 nM Fe′ (Fig. 3a). F_*v*_/F_*m*_ was variable (Fig. 3b), however at Fe′ concentrations ≥ 45.2 nM, the standard deviation of F_*v*_/F_*m*_ was reduced, and the average fluorescence at 120 nM was higher than at 0.01 nM Fe′ (Fig. 3b). Soluble tissue NO_3_^−^ content was greater at 120 nM compared to 1.85–9.56 nM Fe′ (Fig. 3c).

## Discussion

Our finding that growth rates of *M. pyrifera* increased with Fe′ concentration is consistent with studies which found dFe fertilisation increased seaweed biomass^20,23,38,39^. This result supports the suggestion that low dFe concentrations (< 1 nM) associated with deforestation and urbanisation in coastal Japanese waters has caused the disappearance of many kelp species including *Laminaria japonica* and *Undaria pinnatifida* and highlights the importance of dFe for healthy kelp growth^21,23,38,40^. The observed increase in DOC released at concentrations ≤ 20.2 nM Fe′ is comparable to that of oceanic phytoplankton cultured under dFe-limited conditions where DOC is released as an overflow mechanism for photosynthetically fixed organic carbon which cannot be used for growth^30^. DOC release by *M. pyrifera* under limiting Fe′ concentrations (≤ 20.2 nM) may indirectly affect the microbial ecology of the open ocean through stimulating or inhibiting the microbial community with an altered bioavailability of molecules (spanning labile-refractory) compared to the naturally occurring DOC of the open ocean^41,42^.

Iron is essential for electron transport, and the elevated photosynthetic rates at ≥ 45.2 nM Fe′ are likely due to the synthesis of more iron-containing proteins to carry electrons between PSII and PSI, and increased ferredoxin content for NADPH reduction and subsequent oxygen production^43^. Respiration rates were highly correlated with net photosynthesis, indicating that differences in respiration rates were also driven by Fe′ availability (Fig. 2d)^9,43^. C:N ratios were higher at 20.2, 45.2 and 120 nM compared to 0.01 nM Fe′ (Fig. 3d), and this was driven by an increased percent of tissue carbon rather than tissue nitrogen at higher Fe′ concentrations (Supplementary Fig. 4a, b). These results suggest that the additional carbon fixed by *M. pyrifera* at ≥ 45.2 nM Fe′ (indicated by higher photosynthetic rates) could be used for growth and was not released as DOC. Low C:N can be indicative of organic carbon lability^44^ and correlates with the observation that *M. pyrifera* replicates at 0.01–20.2 nM Fe′ were quickly consumed by bacteria—as observed by cell lysis and fragmentation^45^ (Fig. 1a).

The increase in total chlorophyll pigment content ≥ 45.2 nM Fe′ supports other published seaweed, phytoplankton and seagrass research which found a positive correlation between chlorophyll *a* content and increasing dFe concentration—related to a lower content of iron-rich pigment–protein complexes^20,22,26,46–48^. F_*v*_/F_*m*_ results were variable as damaged reaction centres can continue to fluoresce albeit less efficiently^43^. This is consistent with reports that dFe-limited phytoplankton have a greater decrease in PSII and PSI reaction centres compared to antenna pigments, therefore the absorbed excitation energy has a reduced potential of finding a photochemical trap and will likely be re-emitted as fluorescence^18,43^. In eukaryotic phytoplankton, dFe limits NO_3_^−^ acquisition through its role in the photosynthetic pathway rather than through the limitation of NO_3_^−^-reducing enzymes (NO_3_^−^ reductase and NO_2_^−^ reductase)^49^; however, such mechanisms have not been studied in seaweeds. Together our results highlight the importance of Fe′ for healthy kelp growth, and provide useful physiological insights of how limiting Fe′ concentrations (≤ 20.2 nM) decrease the capacity of *M. pyrifera* to photosynthesise and store NO_3_^−^, resulting in an increased production of DOC.

Fe′ appears to be a primary limiting nutrient that restricts kelp from growing in the open ocean and on this basis, *M. pyrifera*—as well as other nearshore kelps such as *Saccharina japonica*^38,39,50^, *Saccharina latissima*^3^ and *Sargassum sp*^1^. being considered for OMA—will not subsist in the open ocean without any nutrient or trace-metal additions. We suggest that large, fleshy seaweeds are mostly confined to coastlines globally due to their high requirement for dFe, the supply of which is more abundant near coastlines. This relationship between the magnitude of dFe requirements and dFe supply has also been observed for coastal phytoplankton^18^. Previous estimates of suitable oceanic ‘real estate’ for kelp aquaculture considered nitrogen and phosphorus availability along with temperature^6^ but did not consider dFe. Our data support early studies on 'oceanic' (~ 2 km from the coastline) kelp aquaculture that ‘irrigated’ *M. pyrifera* using an artificial/ purposeful upwelling system but could not sustain growth with upwelled seawater (~ 300 m depth) without adding dFe^51^.

One departure from the geographical limits placed on seaweeds in the coastal ocean is the success of two species of brown seaweed (order Fucales) from the specious genus S*argassum* (*S. natans* and *S. fluitans*) offshore in the Sargasso Sea, N. Atlantic where they naturally form ‘golden tides’^52^. Their ability to form a significant area of rafts in this region is probably due to a specific set of circumstances. Firstly, their distinctive clonal reproduction by fragmentation (i.e., non-reproductive vegetative growth), a mechanism of reproduction that kelps (order Laminariales) do not undergo^53,54^. Secondly, these *Sargassum* communities subsist in the Sargasso Sea under low dFe conditions (with a total dFe concentration of 0.2–0.8 nM^55^); however, growth is likely sustained by a combination of a high iron storage potential—a unique iron ‘plaque’ on the seaweed cell surface^54^—and most importantly, aeolian inputs of iron from Africa^56^ and the circulatory ocean current which facilitates the (recurring) transport of seaweeds past the higher dFe waters near the North American continent. The latter is evidenced by increasing productivity of both pelagic *Sargassum* species during their passage through North American coastal waters where dFe resupply mechanisms are prevalent and concentrations of dFe will likely be higher^57^.

This multi-stranded mechanism for the unique long-term success of the Sargasso sea macroalgae is supported by another example of an open-ocean seaweed population: the recent (~ 10 years) annually re-occurring trans-basin belts of floating *Sargassum* in the (sub)tropical North Atlantic, known as the Great Atlantic Sargassum Belt (GASB)^58^. The recent emergence of this *Sargassum* population is thought to be driven by increased anthropogenically driven nutrient runoff from the Amazon River, thus the emergence of the GASB is likely related to ocean nutrient, and in particular iron, fertilisation^16,59^, only reinforcing the importance of the re-circulating gyre, and nutrient resupply, in the Sargasso Sea to support offshore *Sargassum* populations^57^.

Consequently, the two case studies, one natural the other anthropogenic, support our conclusion that limiting offshore dFe concentrations will very likely prevent kelp growth in the open ocean—and may prevent OMA— unless there is additional iron fertilisation. The purposeful addition of dFe to offshore waters to stimulate phytoplankton blooms has already raised widespread concerns around multifaceted side-effects^60–62^ along with major unknowns such as its carbon sequestration potential^58,63^. Based on the results of this study, OMA will therefore likely require additional perturbation of offshore waters via dFe fertilisation. The need to implement OMA in conjunction with dFe supply would result in a compound perturbation of open-ocean colonisation by kelps (considered an invasion of the open ocean^41^) and dFe fertilisation. Together, they would increase the many uncertainties both ecological^41^ and biogeochemical^58^ associated with OMA, with implications for open-ocean ecology (competition for added dFe with resident phytoplankton) and gaining social license^62^.

## Methods

### Seawater collection

Trace-metal-clean seawater was obtained from near the Southern Ocean Time Series (SOTS) site located at 47°S and 141°E southwest of Tasmania, Australia on RV *Investigator* voyage IN2019_V02. Seawater was collected from a depth of 12 m using acid-cleaned, Teflon-coated, externally-sprung, 12-litre Niskin bottles attached to an autonomous rosette (SeaBird) equipped with an SBE 911plus CTD unit (SeaBird). Upon retrieval, the Niskin bottles were transferred into a clean container laboratory. Carboys were filled with filtered seawater (0.2 μm, Supor AcroPak 200, Pall), sealed, and covered in plastic to avoid potential trace-metal contamination.

### Clean procedures

Experiment containers (*n* = 48) were constructed using high-density polyethylene (HDPE) bottles (250 mL, Nalgene^®^) with holes drilled in the lids for silicon tubing to pass through for aeration and sampling of seawater. HDPE bottles, LDPE bottles, all-plastic syringes (Thermo Scientific™ Titan3™), silicon tubing, Teflon forceps and PPE clamps used during the dFe experiment were trace metal cleaned following procedures outlined in the ‘Sampling and Sample-handling Protocols for GEOTRACES Cruises’^64^. HDPE sample bottles, low-density polyethylene (LDPE, Nalgene^®^) and silicon tubing were soaked in 2% v/v Decon-90 for one week, rinsed with ultra-high purity water (Milli-Q) four times and submerged in 10% hydrochloric acid (HCl) at 80 °C for one week and finally rinsed with Milli-Q water four times prior to drying inside a laminar flow hood. A trace-metal free, positive pressurised HEPA-filtered air ‘bubble’ was constructed from plastic sheeting and erected in a temperature-controlled room. All sample handling was undertaken within the bubble using trace-metal-clean protocols. The temperature inside the trace-metal free bubble was 13 °C, and water motion in each flask was provided by bubbling filtered air (0.22 μm). Overhead irradiance was provided by cool white fluorescent lights (Thorn Lighting Cadet Batten, HPF) set to 150 μmol photons m^−2^ s^−1^ on a 14:10 light:dark cycle (measured using a LI-COR LI-250 light metre).

Dissolved organic carbon (DOC) vials (Shimadzu TOC) were soaked overnight in 2% v/v Decon-90, rinsed twice in distilled water, washed another night in HCl (ACS reagent, 37%) 10% v/v, rinsed three times in distilled water and—in addition to glass filter paper (Whatman GF/F)—combusted in a furnace overnight to remove any residual carbon. Nutrient tubes were soaked overnight in 2% v/v Decon-90, rinsed twice in distilled water, soaked another night in HCl 10% v/v and finally rinsed three times in distilled water.

### Experimental design

Open-ocean seawater (pH 8.05) was spiked with Aquil medium ethylenediaminetetraacetic acid (EDTA) and nutrient additions using trace-metal-clean techniques so that the final concentrations in flasks were as follows: 100 µM EDTA, 200 μM nitrogen as sodium nitrate (NaNO_3_), 10 μM phosphorus as dibasic sodium phosphate (NaH_2_PO_4_), 0.1 μM Molybdenum (Na_2_MoO_4_•2H_2_O), 0.05 μM Cobalt (CoCl_2_•6H_2_O), 0.0781 μM Zinc (ZnSO_4_•7H_2_O), 0.0198 μM Copper (CuSO_4_•5H_2_O) and 0.456 μM Manganese (MnCl_2_•4H_2_O).

To determine the effect of dFe limitation on *M. pyrifera* physiology, seven treatments with dFe concentrations (added from acidified FeCl_3_ solutions) of 0, 1, 2.5, 5, 10, 20 and 40 μM were set up. Using these dFe concentrations, Fe′ concentrations were calculated for the FeEDTA media following Sunda and Huntsman^65^ at the mean incubation temperature (13 °C), irradiance (150 μmol photons m^−2^ s^−1^, adjusted for the 14:10 h light:dark cycle – I_hv_ = 0.163), and pH 8.05—the mean of the initial ocean seawater. An overall conditional steady-state dissociation constant for FeEDTA chelates of 1.807 × 10^−7^ was used to calculate a Fe′:Fe(tot) ratio of between 1.18 × 10^−3^ at 0.01 nM Fe′ and 2.99 × 10^−3^ at 120 nM Fe′. The overall conditional steady-state dissociation constant (K′(light)) was calculated as the sum of the conditional stability constant in the dark (Kd′ (dark); 4.98 × 10^−8^) and the conditional photo-dissociation constant (Khv; 8.01 × 10^−7^; adjusted for irradiance (I_hv_)) of EDTA at 13 °C. Here we define the Fe′ as the sum of dissolved inorganic Fe(III) species. Defining Fe′ for culture work is important as most incubation experiments involve adding the synthetic chelator EDTA to buffer metal ion concentrations in the culture medium (see Supplementary Fig. 2a–c). The addition of EDTA strongly influences dFe bioavailability. Final Fe′ concentrations were set to simulate the concentration range for biologically available dFe from the open ocean through to the coastal environment. In all except our lowest Fe′ treatments, Fe(oxy)hydroxides formed due to the solubility limit of Fe′ at > 700 pM^32^ and may have been utilised by *M. pyrifera*. Note it was not possible to report Fe′ for the published dFe concentrations in Fig. 1b, as the additional data required for such calculations is not available (see Supplementary Fig. 2). However, a typical range of dFe of 0.1–0.6 nM would equate to an Fe′ of 0.11–1.49 pM (see Supplementary Equation).

Six replicate flasks were enriched to each concentration of Fe′, and four flasks (two with 0.01 nM and two with 120 nM) were used as controls with no added seaweed to ensure results were driven by seaweed not Fe′ addition. The trace-metal ion buffered synthetic seawater medium was sampled for analysis of trace-metal concentrations to determine background concentrations of Fe′ in the seawater prior to enrichment with Fe′. The synthetic seawater samples were acidified for preservation with distilled HCl (Savillex PFA distillation system, DST-1000), triple bagged and stored at 4 °C, until analysis.

### Seaweed collection

*M. pyrifera* apical blades (i.e., first blade divided from the apical meristem, *n* = 42) were collected using snorkel from ~ 1 m depth at Fortescue Bay, Tasmania, Australia (43.13°S, 147.96°E) on October 12, 2021. Seaweeds were placed in an insulated container with seawater for transport to the laboratory 2 h away.

### Experimental procedure and seawater sampling

Each seaweed blade (*n* = 42) was cut into a 5-cm round disk using a plastic cutter and wiped with Kimtech™ wipes to ensure seaweed surface was visibly epiphyte free. Each seaweed disc was an independent replicate. Seaweeds were photographed and weighed for growth measurements (see below), then introduced to experimental flasks in a laminar flow hood. The experiment was run for 14 days, with seawater refreshed and enriched with nutrients every three days to ensure seaweeds were not limited. On day 12, seawater samples were taken from each flask for initial NO_3_^−^ and DOC analysis. Samples were periodically collected using all-plastic syringes (Thermo Scientific™) through silicon tubing. Seawater was sampled at 4 and 8 h after initial sample collection for NO_3_^−^ uptake (depletion from the media) and 24 h after initial sample collection for DOC production. Seawater samples for NO_3_^−^ and DOC production were filtered using pre-combusted Whatman GF/F (0.7 μM) filter paper. NO_3_^−^ seawater samples were stored in 12-mL polyethylene tubes (kept at −20 °C until analysis) and DOC samples were stored in 40-mL glass vials (Shimadzu TOC, preserved with 0.05% Orthophosphoric acid and kept at 4 °C until analysis). On day 13, initial and final photosynthetic measurements were taken as well as initial respiration measurements (see below). On day 14, final respiration measurements were taken, and seaweeds were removed from flasks for final weight, F_*v*_/F_*m*_, ETR and surface area measurements then preserved for the following biological assays.

### Growth rate

Images were taken of each replicate before (day 0) and after the experiment (day 14) using a light board (A3 LED light pad) for analysis of surface area using the image processing programme, ImageJ. Growth rates were calculated using the following equation:

1 $$({{SA}}_{{{{{{\rm{F}}}}}}}-{{SA}}_{{{{{{\rm{I}}}}}}})/{{{{{\rm{d}}}}}}$$

where SA_F_ is the surface area (cm^2^) of the seaweed at the end of the 2-week experiment, SA_I_ is the surface area (cm^2^) of the seaweed at the start of the 2-week experiment, and d is number of experiment days (14).

### F_*v*_/F_*m*_

Pulse amplitude modulated (PAM) fluorometry was used to determine the maximum photochemical efficiency of PSII (F_*v*_/F_*m*,_ where F_*v*_ = F_*m*_ – F_*o*_ and F_*o*_ and F_*m*_, are the minimum and maximum fluorescence in the dark-acclimated state, respectively). F_*v*_/F_*m*_ of all replicates was measured prior to (day 0) and at the conclusion of the experiment (day 14) using a JUNIOR-PAM^TM^ chlorophyll fluorometer (Heinz Walz GmbH, Germany), and individuals were dark-acclimated for 15 min prior to F_*v*_/F_*m*_ measurement.

### DOC seawater analysis

DOC samples were analysed using an automated total organic carbon analyser (Analytica Jena Multi N/C 3100) via combustion at 720 °C over a platinum catalyst in accordance with method 5310 D^66^. Net DOC release rates were based on the rate of change in concentration between initial and final samples and were normalised to flask volume, incubation period, and seaweed biomass (gDW^−1^). Positive rates of net DOC production indicate DOC release, while negative values indicate net DOC uptake in the system.

### Trace-metal seawater analysis

Background dFe concentrations in the oceanic seawater, and media-spiked seawater, were determined using the isotope dilution (ID) technique using enriched isotopes (^57^Fe, ^67^Zn, ^65^Cu, ^61^Ni, ^110^Cd and ^206^Pb), or by the method of standard additions (Mn and Co). The trace metals in the seawater media were preconcentrated on the Nobias Chelate PA1 resin using a home-built preconcentration system. Prior to preconcentration, spiked samples were left for 24 h and then buffered to a pH of around 5.2. Metals were eluted from the Nobias resin with 1 mol L^−1^ nitric acid and then determined by ICPMS (iCap, Thermo Scientific) in helium Kinetic Energy Discrimination (He KED) mode to remove argon oxide inferences on iron. The background Fe′ concentrations in nutrient-spiked seawater were 0.01 nM.

### Soluble tissue nitrogen

Soluble tissue NO_3_^−^ content was analysed following the boiling extraction method Hurd et al.^67^ using fresh seaweed tissue after the completion of the incubation experiment. Boiling tubes were filled with 20 mL of distilled water and 0.2 ± 0.05 g pieces of alga tissue were added. Tubes were removed after overnight refrigeration, capped in aluminium foil and placed in a boiling water bath (~ 100 °C) for 40 min. The solution was left to cool before filtration (Whatman, GF/F) and stored at –20 °C. Boiling extraction was repeated twice to ensure all soluble nitrogen was removed from the alga tissue. The subsequent extract was analysed for concentrations of N(NO_3_^−^ + nitrite (NO_2_^−^)) using a QuickChem® 8000 automated Ion Analyser (LaChat Instruments). Results were standardised to wet weight using the following formula:

2 $$\frac{\left({{{{{{\rm{N}}}}}}}_{1}+{{{{{{\rm{N}}}}}}}_{2}\right)\times 0.02\,}{{{{{{\rm{WW}}}}}}}$$

where N_1_, N_2_ are the N(NO_3_^−^ + NO_2_^−^) concentrations (µM) in the supernatant after the first and second extractions, 0.02 is the volume of liquid in each boiling tube (L), and WW is the wet weight (g) of the seaweed.

### Photosynthesis and respiration rates

On day 13, net photosynthesis was measured (i.e., oxygen (O_2_) evolution, μmol O_2_ produced gWW^−1^ h^−1^) within each culture flask over 3 h under the experimental irradiance (150 μmol photons m^−2^ s^−1^). Respiration (i.e., μmol O_2_ consumed gWW^−1^ h^−1^) was measured for 12 h overnight in the dark. Water motion was provided by an orbital shaker (100 rpm, RATEK). Net photosynthetic measurements were made between 8:00 and 13:00, and respiration measurements between 18:00 and 09:00 + 1 day. Dissolved O_2_ measurements were made with a portable oxygen metre and optical probe (PreSens Fibox 4 trace with OXYPro® Series probe).

### Pigment content

Pigment content (Chlorophyll *a* + Chlorophyll *c*) was determined following methods outlined in refs. ^68–70^ using 0.1 g frozen tissue, preserved at −80 °C after the completion of the incubation experiment. Seaweed pieces (0.1 gWW ± 0.05) were placed in individual centrifuge tubes (15 mL) with 4 ml of dimethyl sulfoxide (DMSO) and left to extract for 15 min. Seaweed pieces were then removed from the DMSO solution and placed in separate tubes with 90% acetone (v/v) and left to extract until algae pieces were colourless (~ 30 min). The absorbance of extracts were measured with a S-22 UV/Vis Spectrophotometer (Halo RB-10, Dynamica Scientific Ltd.), DMSO extract at wavelengths 665, 631, 582, and 480 nm and acetone at 664, 631, 581 and 470 nm. Pigment content was subsequently determined using the equations given by Seely et al.^68–70^.

### %C, %N and C:N ratio

The percent tissue content of carbon (C) and nitrogen (N), and the C:N ratio were determined using a NA1500 elemental analyser coupled to a Thermo Scientific Delta V Plus via a Conflo IV using 0.05 g of oven-dried (60 °C, three nights) seaweed tissue. Combustion and oxidation were achieved at 1020 °C and reduction at 650 °C, respectively, in a column packed with chromium oxide (Cr_2_O_3_), copper oxide (CuO) and silvered cobaltous oxide^71^. Organic carbon and nitrogen contents were determined by comparison of instrument response (area) calibrated using standards with known carbon and nitrogen content. Tissue C and N content were expressed as a percentage of the alga’s dry weight and C:N ratios were based on their atomic weights.

### Statistics and reproducibility

Statistical analysis was conducted using R version 2.2 (R Development Core Team, 2012), and graphs were created using SigmaPlot (Systat Software Inc). Results are shown as boxplots indicating minimum, 1^st^ quartile, median, 3^rd^ quartile and maximum data values (*n* = 6). ANOVA test assumptions were assessed using diagnostic plots of model residuals and data were transformed where necessary using the package, bestNormalize. To compare the parameters for growth, DOC production, photosynthesis, respiration, pigments, F_*v*_/F_*m*_ and soluble tissue NO_3_^−^ with Fe′ concentration, one-way ANOVAs were run. Where statistical significance was observed (*P* < 0.05), a Tukey’s HSD multiple comparison test was run to distinguish which means were statistically significant from others and significance was marked on our plots using letters assigned by a multiple comparison boxplot. A Michaelis–Menten rectangular hyperbola best fit the growth results using the equation:

3 $${{{{{\rm{V}}}}}}={{{{{{\rm{V}}}}}}}_{{{\max }}}\frac{{{{{{\rm{S}}}}}}}{{{{{{{\rm{K}}}}}}}_{{{{{{\rm{m}}}}}}}+{{{{{\rm{S}}}}}}}$$

where V is the increase in growth, V_max_ is the maximum growth, S is the concentration of the dFe, and K_m_ is the half-saturation constant. The rectangular hyperbola was fitted to growth data using SigmaPlot.

### Reporting summary

Further information on research design is available in the Nature Portfolio Reporting Summary linked to this article.

## Supplementary information

Supplementary Information-New

Description of Additional Supplementary Files

Supplementary Data 1

Supplementary Data 2-3

Reporting Summary

## Data Availability

The datasets generated during the current study are available from the corresponding author on reasonable request. Numerical sources for Figs. 2 and  3 are available in Supplementary Data 1.
