# Supplementary material for: Iron limitation of kelp growth may prevent ocean afforestation
Source: Commun Biol. 2023 Jun 6;6:607. doi: 10.1038/s42003-023-04962-4 (PMC10244339; doi:10.1038/s42003-023-04962-4)
Supplement: Supplementary file 1 — Supplementary Information-New [file 42003_2023_4962_MOESM1_ESM.pdf]

Supplementary Material

Equation:

Calculation of Fe(III)′ values for dissolved iron (dFe) concentrations for a typical range (0.1-0.6 nM).

Using the following conditions:

Ligand concentration = 1 nM

Log KFeL = 10^22

Alpha Fe′/Fe3+ = 10^10

The ligand concentration and KFeL values are based on those reported by Boye et al. (2001)[1] for the Southern Ocean.

The following quadratic equation can then be solved:

$$[\mathrm{Fe}^{3+}]^2 \alpha_{\mathrm{Fe}} K'_{\mathrm{FeL}} + [\mathrm{Fe}^{3+}](\alpha_{\mathrm{Fe}} + K'_{\mathrm{FeL}} C_{\mathrm{L}} - K'_{\mathrm{FeL}} C_{\mathrm{Fe}}) - C_{\mathrm{Fe}} = 0.$$

using

$$x = \frac{-b \pm \sqrt{b^2 - 4ac}}{2a}$$

The calculated range is 0.11-1.49 pM Fe′.

Figures:

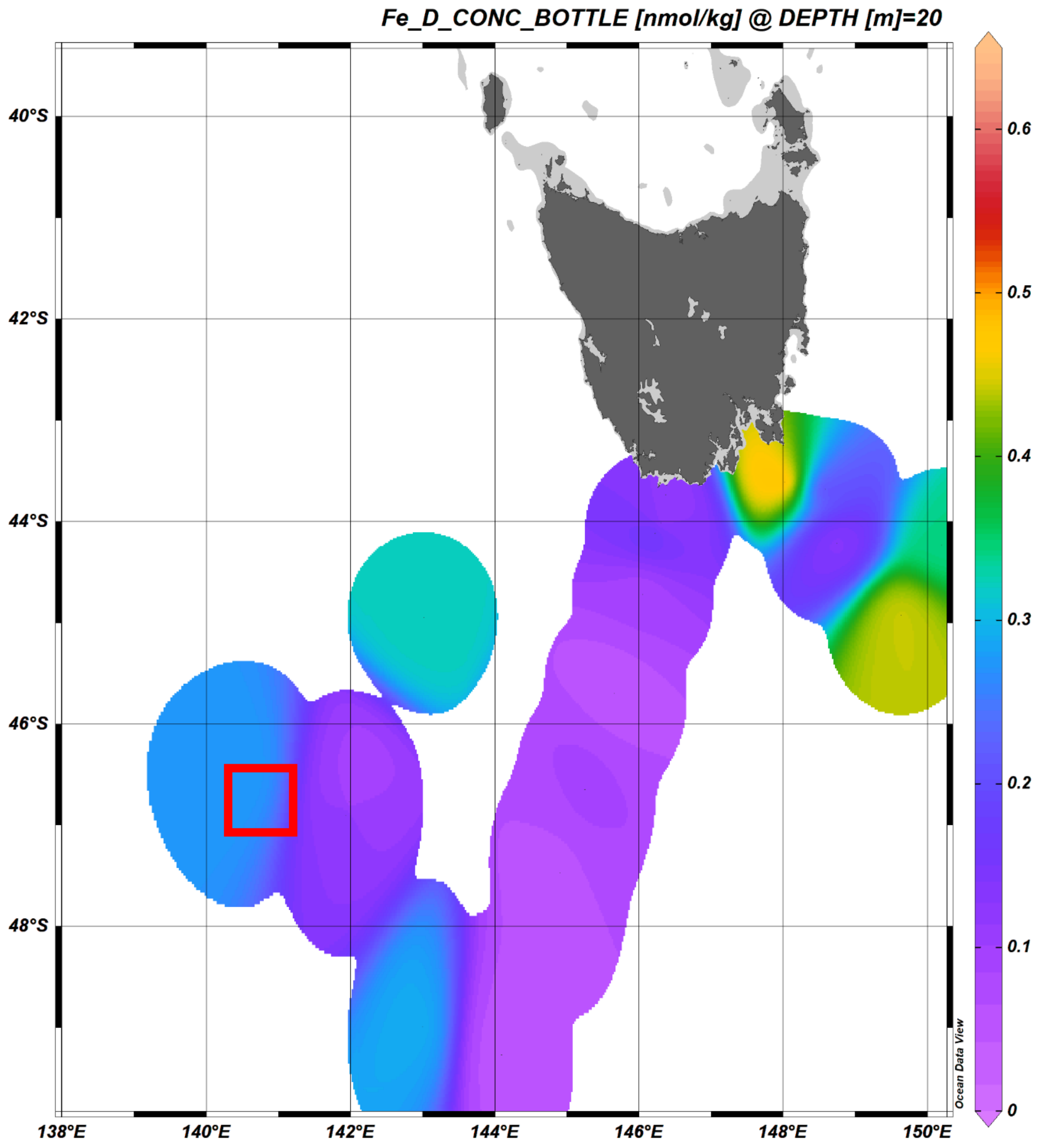

Supplementary Fig. 1 | Location of seawater sampled for the experiment in the Southern Ocean (red box, ~47°S, 141°E) underlaid with interpolated dFe concentrations at 20 m depth using regional datasets within the GEOTRACES Intermediate Data Product (IDP; map created using Ocean Data View, GEOTRACES 2021 IDP2021, DOI: 10.5285/cf2d9ba9-d51d-3b7c-e053-8486abc0f5fd).

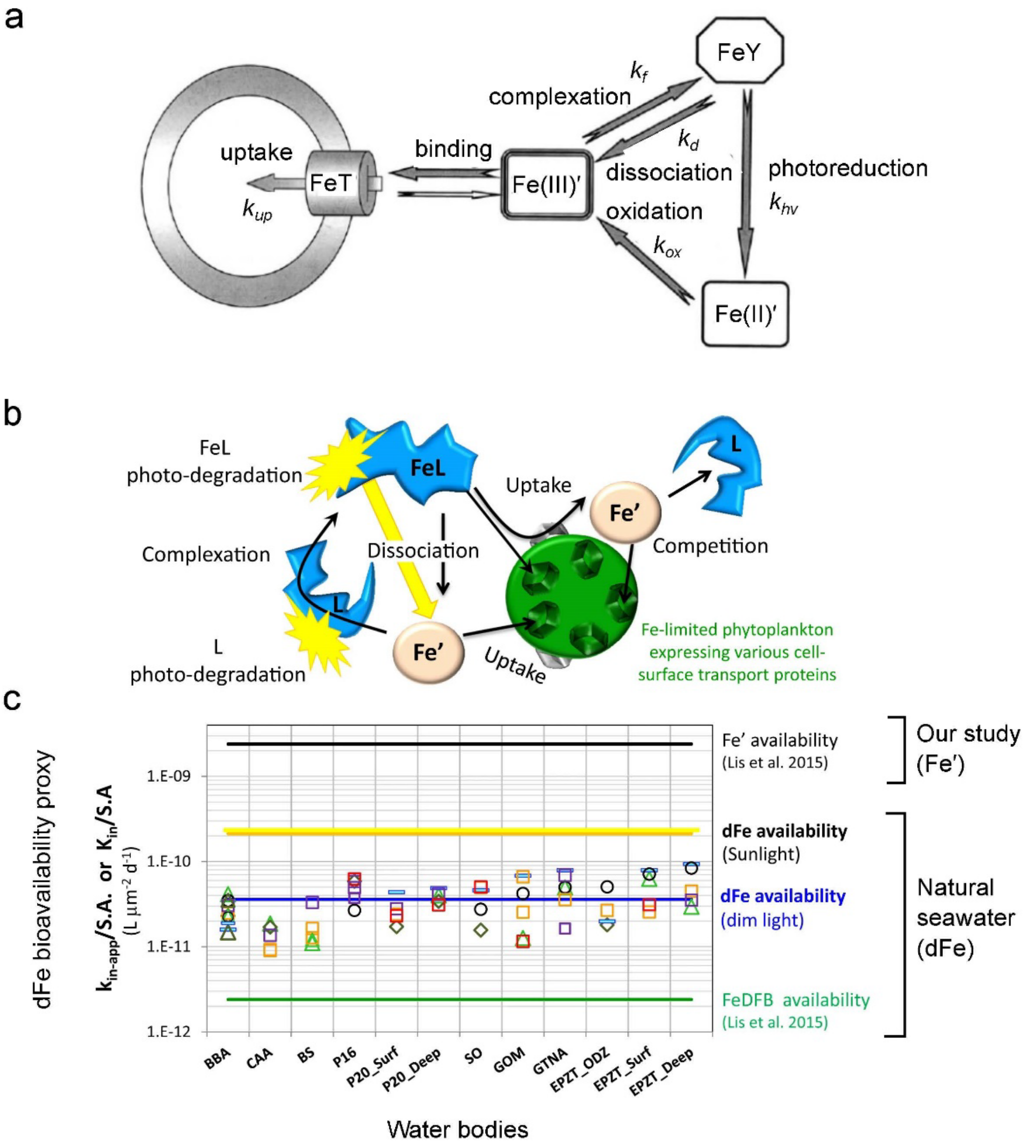

Supplementary Fig. 2 | Conceptual models of the relationship between dFe and Fe′. (a) Schematic illustrations of the Fe′ model. In aqueous media buffered by a large excess of chelating agent Y **(in our experiment Y = EDTA)**, unchelated Fe(III) (Fe′) is maintained at chemical equilibrium with the chelate FeY through dissociation (rate constant k_d) and complexation (rate constant k_f) reactions. Upon illumination, photoreduction of photolabile FeY generates unchelated reduced iron, Fe(II)′, which is rapidly oxidized to Fe(III)′. Fe(III)′ is the dominant species that binds to cell surface ligand (FeT) and is being transported across the cell membrane**.** The rate of uptake is proportional to Fe(III)′ concentrations (rate constant k_{up}). **For simplicity, we refer to Fe(III)′ as Fe′ in our manuscript.** Adapted from Shaked et al. (2005)[2]**.** (b) Schematic representation of the major reactions at play in seawater, which govern the rate of uptake of iron (Fe) from inorganic (Fe′) and organic (FeL) complexes by

Fe-limited phytoplankton, and subsequently determine the dFe bioavailability proxy—k_{in-app}/S.A. Adapted from Shaked et al. (2020)[3], and (c) summary of all data evaluating dFe bioavailability across all water bodies from Shaked et al. (2020)[3] in dim laboratory light and in outdoor full light, plotted within the framework of the bioavailability envelope of Lis et al. (2015)[4]. See original article for water body descriptions and phytoplankton strains. Permissions: Authors acquired permission from Professor Yeala Shaked, (Hebrew University, Israel) the original creator of all three figure panels used in Supplementary Figure Two.

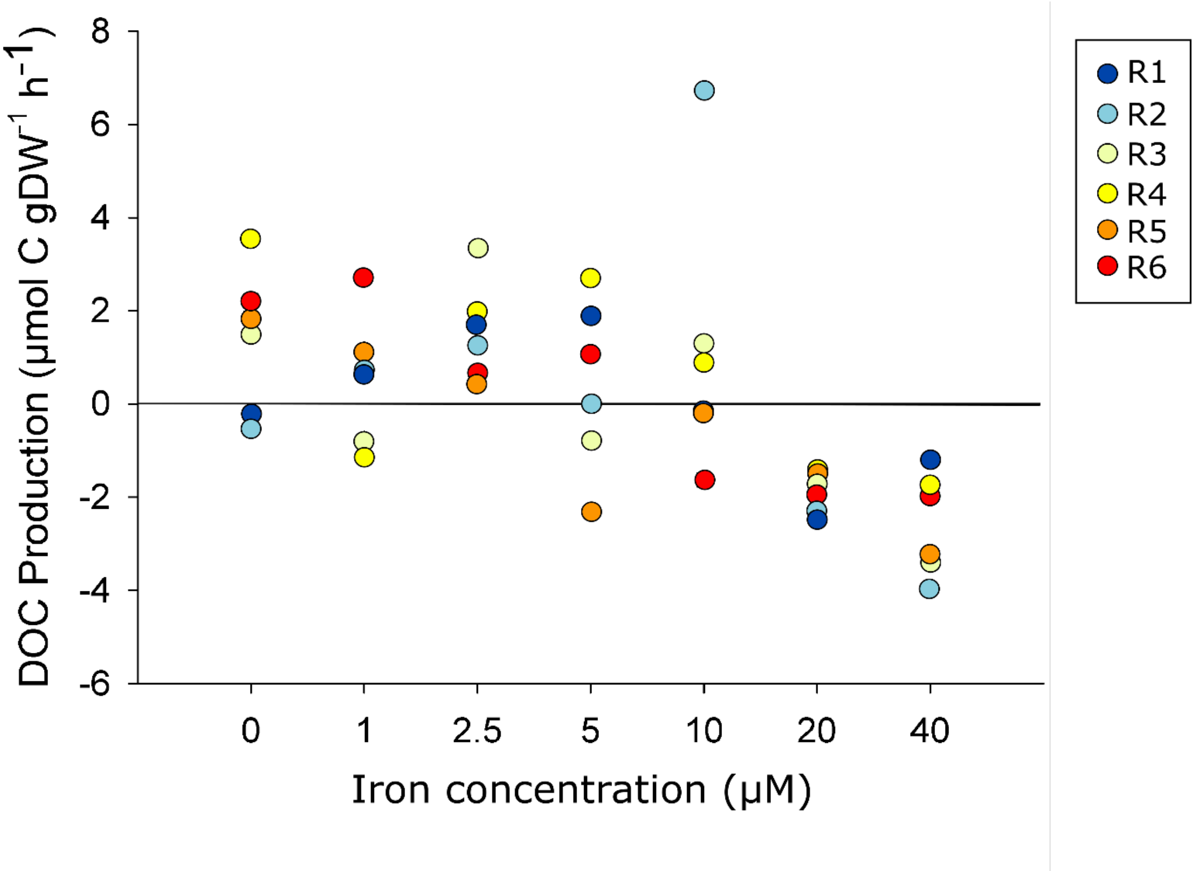

Supplementary Fig. 3 | Production of dissolved organic carbon (DOC) by individual replicate disks of *M. pyrifera* (see Fig.1a, n = 6 labelled R1-R6). Higher rates are observed for those with tissue disintegration, indicating that fragmentation of kelp blades enhances DOC release. The gradient of dFe concentrations (added from acidified $FeCl_3$ solutions) of 0, 1, 2.5, 5, 10, 20 and 40 µM equate to Fe′ concentrations, 0.01, 1.85, 4.67, 9.56, 20.15, 45.18 and 119.85 nM.

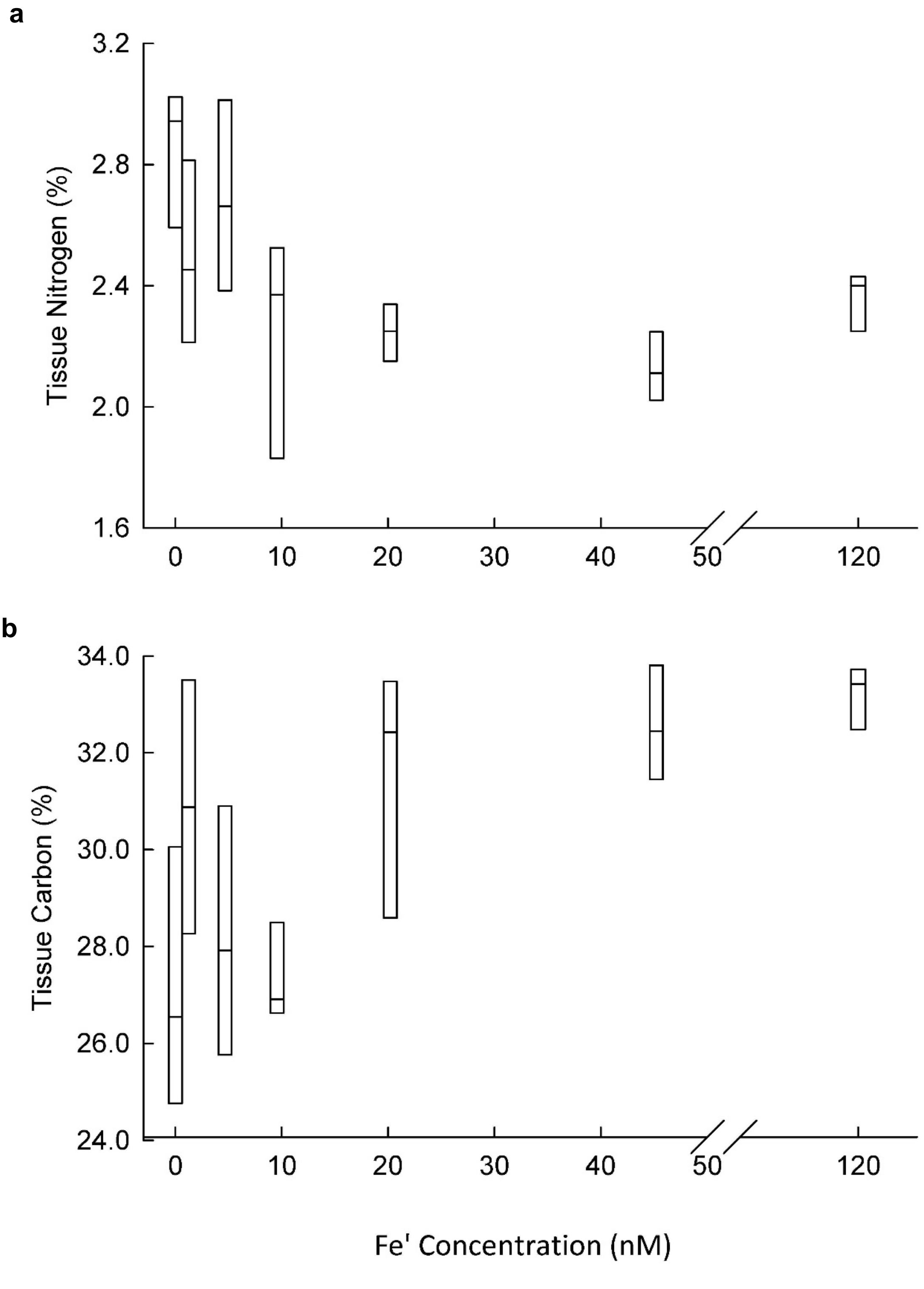

Supplementary Fig. 4 | (a) Percent dry tissue nitrogen content and (b) percent dry tissue carbon for *M. pyrifera* grown at each of a range of seawater dFe concentrations. Boxes show minimum, 1st quartile, median, 3rd quartile and maximum data values (n = 6).

Supplementary Material References

1. Boye, M., van den Berg, C. M. G., de Jong, J. T. M., Leach, H., Croot, P. & de Baar, H. J. W. 2001. Organic complexation of iron in the Southern Ocean. *Deep Sea Research Part I: Oceanographic Research Papers* 48:1477-97.

2. Shaked, Y., Kustka, A. B. & Morel, F. M. M. 2005. A general kinetic model for iron aquisition by eukaryotic phytoplankton. *Limnology and Oceanography* 50:872-82.

3. Shaked, Y., Buck, K. N., Mellett, T. & Maldonado, M. T. 2020. Insights into the bioavailability of oceanic dissolved Fe from phytoplankton uptake kinetics. *The ISME journal* 14:1182-93.

4. Lis, H., Shaked, Y., Kranzler, C., Keren, N. & Morel, F. M. M. 2015. Iron bioavailability to phytoplankton: an empirical approach. *The ISME journal* 9:1003-13.
