# Supplementary material for: Iron limitation of kelp growth may prevent ocean afforestation
Source: Commun Biol. 2023 Jun 6;6:607. doi: 10.1038/s42003-023-04962-4 (PMC10244339; doi:10.1038/s42003-023-04962-4)
Supplement: Supplementary file 2 — Description of Additional Supplementary Files [file 42003_2023_4962_MOESM2_ESM.pdf]

## Description of Additional Supplementary Files

**File name:** Supplementary Data 1

**Description:** ANOVA Test Results.

**File name:** Supplementary Data 2

**Description:** Tukey's HSD Multiple Comparison Results.

**File name:** Supplementary Data 3

**Description:** The source data behind the graphs in the paper.
