# Supplementary material for: Iron limitation of kelp growth may prevent ocean afforestation
Source: Commun Biol. 2023 Jun 6;6:607. doi: 10.1038/s42003-023-04962-4 (PMC10244339; doi:10.1038/s42003-023-04962-4)
Supplement: Supplementary file 5 — Reporting Summary [file 42003_2023_4962_MOESM5_ESM.pdf]

nature portfolio

Corresponding author(s): Ellie Paine

Last updated by author(s): 07/02/2023

# Reporting Summary

Nature Portfolio wishes to improve the reproducibility of the work that we publish. This form provides structure for consistency and transparency in reporting. For further information on Nature Portfolio policies, see our Editorial Policies and the Editorial Policy Checklist.

## Statistics

For all statistical analyses, confirm that the following items are present in the figure legend, table legend, main text, or Methods section.

| n/a | Confirmed | |
|---|---|---|
| ☐ | ☒ | The exact sample size (*n*) for each experimental group/condition, given as a discrete number and unit of measurement |
| ☐ | ☒ | A statement on whether measurements were taken from distinct samples or whether the same sample was measured repeatedly |
| ☐ | ☒ | The statistical test(s) used AND whether they are one- or two-sided<br>*Only common tests should be described solely by name; describe more complex techniques in the Methods section.* |
| ☐ | ☒ | A description of all covariates tested |
| ☐ | ☒ | A description of any assumptions or corrections, such as tests of normality and adjustment for multiple comparisons |
| ☐ | ☒ | A full description of the statistical parameters including central tendency (e.g. means) or other basic estimates (e.g. regression coefficient) AND variation (e.g. standard deviation) or associated estimates of uncertainty (e.g. confidence intervals) |
| ☐ | ☒ | For null hypothesis testing, the test statistic (e.g. *F*, *t*, *r*) with confidence intervals, effect sizes, degrees of freedom and *P* value noted<br>*Give P values as exact values whenever suitable.* |
| ☒ | ☐ | For Bayesian analysis, information on the choice of priors and Markov chain Monte Carlo settings |
| ☒ | ☐ | For hierarchical and complex designs, identification of the appropriate level for tests and full reporting of outcomes |
| ☒ | ☐ | Estimates of effect sizes (e.g. Cohen's *d*, Pearson's *r*), indicating how they were calculated |

*Our web collection on statistics for biologists contains articles on many of the points above.*

## Software and code

Policy information about availability of computer code

| | |
|---|---|
| Data collection | Data was obtained from laboratory physiological assays of seaweed tissue. Statistical analysis was conducted using R version 2.2 (R Development Core Team, 2012) |
| Data analysis | Statistical analysis was conducted using R version 2.2 (R Development Core Team, 2012) |

For manuscripts utilizing custom algorithms or software that are central to the research but not yet described in published literature, software must be made available to editors and reviewers. We strongly encourage code deposition in a community repository (e.g. GitHub). See the Nature Portfolio guidelines for submitting code & software for further information.

## Data

Policy information about availability of data

All manuscripts must include a data availability statement. This statement should provide the following information, where applicable:

- Accession codes, unique identifiers, or web links for publicly available datasets
- A description of any restrictions on data availability
- For clinical datasets or third party data, please ensure that the statement adheres to our policy

The datasets generated during the current study are available from the corresponding author on reasonable request.

## Human research participants

Policy information about studies involving human research participants and Sex and Gender in Research.

| | |
|---|---|
| Reporting on sex and gender | N/A |
| Population characteristics | N/A |
| Recruitment | N/A |
| Ethics oversight | N/A |

Note that full information on the approval of the study protocol must also be provided in the manuscript.

# Field-specific reporting

Please select the one below that is the best fit for your research. If you are not sure, read the appropriate sections before making your selection.

☐ Life sciences ☐ Behavioural & social sciences ☒ Ecological, evolutionary & environmental sciences

For a reference copy of the document with all sections, see nature.com/documents/nr-reporting-summary-flat.pdf

# Ecological, evolutionary & environmental sciences study design

All studies must disclose on these points even when the disclosure is negative.

| | |
|---|---|
| Study description | We determined the limiting dissolved iron concentrations for growth and key physiological functions of a kelp species, Macrocystis pyrifera. |
| Research sample | 42 individual seaweed blades (each from a separate individual seaweed) were collected for use in this laboratory experiment. 6 replicate blades were used for each concentration of iron. |
| Sampling strategy | Sample size of 6 per iron treatment (7 iron treatments therefore, 42 total) is appropriate for physiological experiments. |
| Data collection | Experimental data collected in a laboratory. |
| Timing and spatial scale | Iron incubation experiment ran for 2 weeks. |
| Data exclusions | No data were excluded from the analyses. |
| Reproducibility | Methods and results are considered reproducible. |
| Randomization | Individual seaweed replicates were randomly assigned to each iron treatment. |
| Blinding | Not relevant |

Did the study involve field work? ☒ Yes ☐ No

## Field work, collection and transport

| | |
|---|---|
| Field conditions | Collection of seaweed blades from Fortescue Bay in Tasmania. |
| Location | M. pyrifera apical blades (i.e., first blade divided from the apical meristem, n = 42) were collected using snorkel from ~1 m depth at Fortescue Bay, Tasmania, Australia (43.13° S, 147.96° E) on the 12th of October 2021 |
| Access & import/export | Tasmanian Government Permit was used for the snorkel collection of seaweeds (number: 20097). |
| Disturbance | Only one blade was sampled per individual seaweed. |

# Reporting for specific materials, systems and methods

We require information from authors about some types of materials, experimental systems and methods used in many studies. Here, indicate whether each material, system or method listed is relevant to your study. If you are not sure if a list item applies to your research, read the appropriate section before selecting a response.

## Materials & experimental systems

| n/a | Involved in the study |
| --- | --- |
| ☒ | ☐ Antibodies |
| ☒ | ☐ Eukaryotic cell lines |
| ☒ | ☐ Palaeontology and archaeology |
| ☒ | ☐ Animals and other organisms |
| ☒ | ☐ Clinical data |
| ☒ | ☐ Dual use research of concern |

## Methods

| n/a | Involved in the study |
| --- | --- |
| ☒ | ☐ ChIP-seq |
| ☒ | ☐ Flow cytometry |
| ☒ | ☐ MRI-based neuroimaging |
